# Right-Sided Epididymo-Orchitis as a Presentation of Diverticular Colovesical Fistula

**DOI:** 10.7759/cureus.35376

**Published:** 2023-02-23

**Authors:** Kavina Sidhu, Doruk Seyfi, Christopher Byrne

**Affiliations:** 1 Colorectal Surgery, Royal Prince Alfred Hospital, Sydney, AUS

**Keywords:** fecaluria, epididymo-orchitis, diverticulitis, laparoscopic anterior resection, intravesical gas, urosepsis, pneumaturia, complicated diverticulitis, recurrent urinary tract infections, colovesical fistula

## Abstract

A colovesical fistula is a recognized complication of diverticulitis. Although the underlying pathology is usually of colonic origin, the majority of patients present with urological symptoms, classically pneumaturia, and urinary tract infection. Epididymo-orchitis is a rare presentation. It is important to identify elderly males who present with recurrent urosepsis and/or epididymo-orchitis refractory to medical treatment as they may have an underlying benign or malignant etiology. The diagnostic challenge in these cases is to confirm the presence of a fistula, exclude malignancy, and determine the underlying pathology.

We present a case of diverticular colovesical fistula in an elderly male who presented with symptoms of epididymo-orchitis on a background of recurrent urinary tract infections. The presence of intravesical gas within the left posterolateral bladder wall and soft tissue thickening continuous with the mid-sigmoid colon was consistent with a colovesical fistula. This patient underwent elective laparoscopic anterior resection and repair of colovesical fistula.

## Introduction

A colovesical fistula is an abnormal communication between the large bowel and the bladder, and it accounts for 1:3000 surgical admissions with a male-to-female ratio of 3:1 [1,2]. It is a rare complication of inflammatory or neoplastic disease and occasionally results from trauma, iatrogenic injuries, or pelvic irradiation. Diverticulitis is more prevalent in the aging population and contributes to 50-70% of cases although the relative risk of developing a colovesical fistula in diverticulitis is 1-4% [1]. The most common tract is usually between the sigmoid colon and bladder dome (62%) caused by direct extension of a ruptured diverticulum or erosion of a peri-diverticular abscess into the bladder [3].

This case discusses persistent urosepsis in the setting of a colovesical fistula presenting as epididymo-orchitis. Exclusion of diverticular colovesical fistula is important especially in older patients presenting with recurrent episodes of urinary tract infections or epididymo-orchitis.

## Case presentation

A male in his 80s presented with right groin and testicular pain. A week prior, he completed a course of cephalexin for a urinary tract infection with partial improvement in his chronic lower urinary tract symptoms. He was otherwise well but had previous open bilateral inguinal hernia repair.

He had elevated inflammatory markers (white cell count: 11x10^9^/L; c-reactive protein: 84 mg/L). Urinalysis demonstrated sterile pyuria and the sexually transmitted infections (STI) screen was unremarkable. He had a mildly distended, soft abdomen with right lower quadrant discomfort and right groin tenderness. Mild scrotal erythema and a high-riding tender, firm right testis with thickened cord structures were noted.

**Figure 1:**
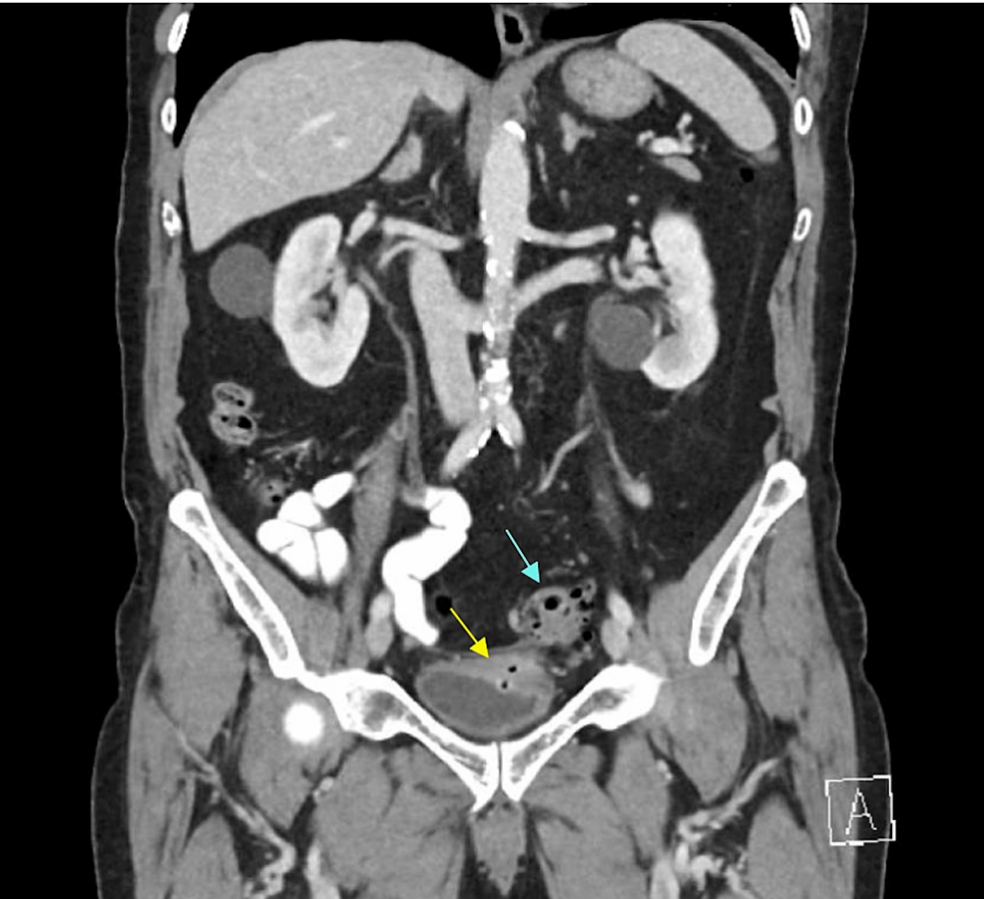
Coronal computed tomography of the abdomen and pelvis with intravenous and oral contrast (Gastrografin). The image shows gas locules within the bladder (yellow arrow) and adjacent sigmoid diverticulitis (blue arrow) consistent with colovesical fistula.

**Figure 2:**
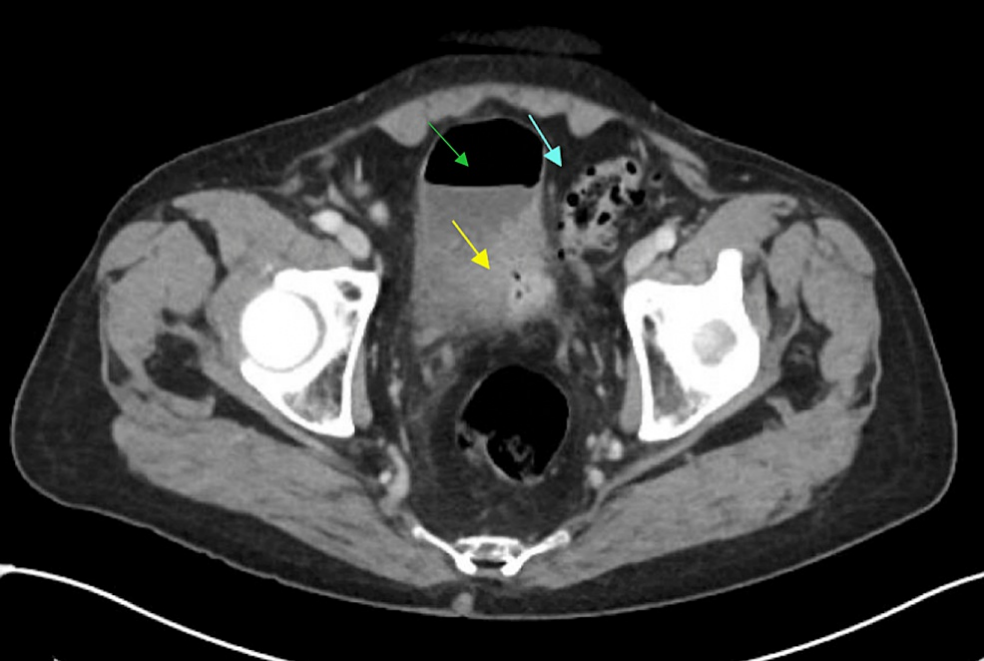
Axial computed tomography of the abdomen and pelvis with intravenous and oral contrast (Gastrografin). Gas locules (yellow arrow) and anti-dependent gas (green arrow) within the bladder and adjacent sigmoid diverticulitis (blue arrow) consistent with colovesical fistula.

Computed tomography of the abdomen and pelvis demonstrated thickening of the left posterolateral bladder wall with multiple gas locules and soft tissue thickening continuous with the sigmoid colon reflecting diverticulitis (Figures 1, 2). The bladder contained anti-dependent gas suspicious for a colovesical fistula (Figure 2).

**Figure 3:**
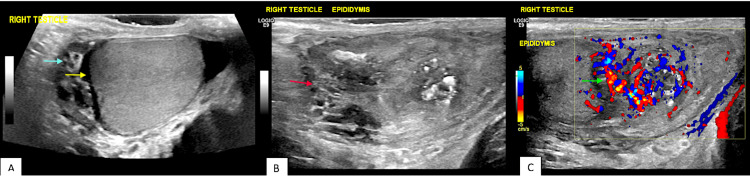
Ultrasound images of the right testicle and epididymis demonstrating acute right epididymitis and mild orchitis. (A) Right testicle and small right hydrocele (yellow arrow) with low-level internal echoes and separations (blue arrow). (B) Swollen, edematous right epididymis (red arrow). (C) Markedly hyperemic and increased vascularity (green arrow) in right epididymis.

A scrotal ultrasound revealed an oedematous, markedly hyperaemic right epididymis in keeping with acute epididymo-orchitis and a reactive hydrocele (Figures 3A-3C).

The patient had a normal colonoscopy five years earlier. A diagnosis of sigmoid diverticulitis, colovesical fistula, and right epididymo-orchitis was made. He was admitted to hospital for further investigation and non-operative management including intravenous antibiotics, analgesia, and venous thromboembolism prophylaxis.

A few days later, flexible sigmoidoscopy revealed a stricture at 30 cm which the scope was unable to traverse without presence of diverticula. Cystoscopy demonstrated mild bladder trabeculations with cystitis at the left posterior wall over a 4 cm area. Biopsies of the posterior bladder wall demonstrated mild-to-moderate cystitis with normal overlying urothelium. No evidence of neoplasm or fistula tract was seen.

He was discharged on two weeks of oral antibiotics. He later experienced pneumaturia and fecaluria. Two weeks later, he underwent elective laparoscopic anterior resection and repair of colovesical fistula (bowel transected proximal to diverticular abscess/fistula site in healthy bowel, end-to-side anastomosis performed and colonoscope passed up to hepatic flexure which was normal). No surgical intervention was performed for the bladder defect. A urinary catheter was kept in situ for a few days after surgery. The post-operative fluoroscopic cystogram and computed tomography did not demonstrate any leak from the urinary tract and colonic anastomosis, respectively. Recovery was complicated by small bowel obstruction secondary to a drain site hernia requiring surgical repair on day five - small knuckle of bowel adherent to fascia herniated through the 10 mm right inferior fossa drain site. Incision was enlarged slightly, viable bowel reduced, and defect sutured (three 1.0 Nylon figure of eight sutures to fascia, 2.0 Vicryl fat closure, and 3.0 Monocryl for skin closure).

**Figure 4:**
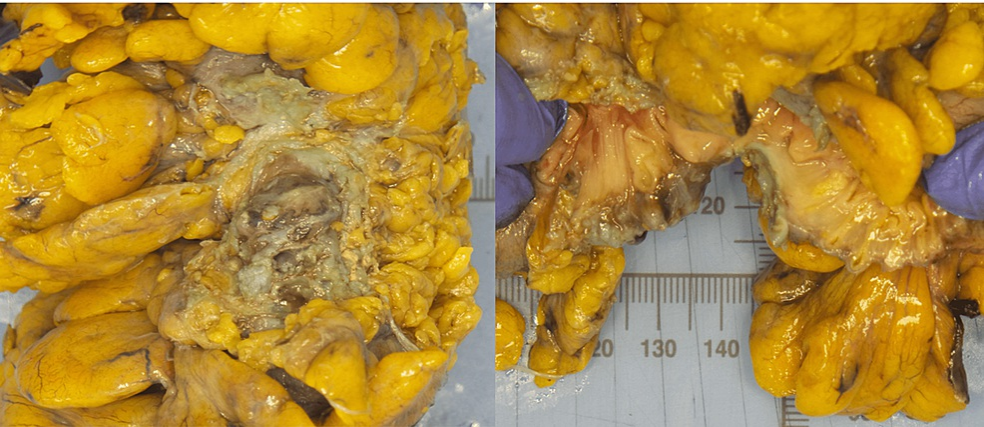
Macroscopic pathology of the patient. Area of fibrous adhesion on serosal surface of bowel wall. Multiple diverticula and intramural abscess are also seen.

An outpatient appointment was arranged six weeks following surgery. The patient recovered well and returned to normal activities. Histopathology revealed diverticular disease with intramural abscess (Figure 4).

## Discussion

The underlying pathology of colovesical fistula is usually of colonic origin, however, the majority of patients present with urological symptoms. Recurrent urinary tract infections with intestinal organisms occur in 57-75% of cases presenting with Gouverneur’s syndrome (suprapubic pain, frequency, dysuria, and tenesmus) [1-4]. Pneumaturia (50%) or fecaluria (40%) are pathognomonic features, delaying diagnosis when absent [4].

Epididymo-orchitis is a rare presentation caused by retrograde passage of bacteria from the prostatic urethra to the epididymis via the ejaculatory ducts and vas deferens. Only a few cases have been reported to date - a case of direct seminal vesicle fistula caused by sigmoid diverticulitis resulting in bladder irritability and emphysematous epididymitis; and a case of urethritis secondary to colovesical fistula [4].

The diagnostic challenge in these cases is to confirm the presence of a fistula, exclude malignancy and determine the underlying pathology. Only 42% of bladder fistulae were correctly diagnosed before admission in one large series and communication demonstrated in only 38% in another [5]. Computed tomography is the main diagnostic tool with sensitivity of 60-100% [6]. It can demonstrate the presence of intravesical gas, passage of oral or rectal contrast into the bladder, colonic wall thickening adjacent to an area of locally thickened bladder wall, and/or extraluminal mass containing air. It is essential in surgical planning and determining timing for surgery (approximately four weeks post inflammation) as it also allows assessment of the severity of inflammation and other complications of acute diverticulitis, e.g., abscess or collection. Although magnetic resonance imaging (MRI) is most accurate and highly sensitive, its role is limited by availability and cost [1]. MRI provides excellent intrinsic soft tissue resolution without requiring contrast. Fluid or air within the fistula tract acts as natural luminal contrast delineating the extension of the fistula relative to the adjacent hollow viscera and demonstrating inflammatory changes in the fat planes.

Endoscopic methods are routine investigative tools to directly visualize the fistulous tract. Cystoscopy has a sensitivity of 38-48% and is essential to exclude urological malignancy [7,8]. It usually demonstrates changes suggestive of a fistula (localized area of erythema or edema) rather than the actual opening itself. Colonoscopy is vital to exclude bowel malignancy and determine the underlying pathology.

Conservative treatment is usually reserved for poor surgical candidates or minimally symptomatic patients with non-malignant colovesical fistula [1]. Classically, the treatment was a defunctioning colostomy proposed by de Melle et al. in 1843, reserved for patients with large volume fistula and poor prognosis [3]. Spontaneous fistula closure occurs in approximately 2% of patients [6]. Small studies (Amin et al. and Solkar et al.) have discussed conservative management with little inconvenience and without significant complication in individualized patients with close follow-up [8]. No randomized controlled trials have supported conservative or operative management and only one case report demonstrated complete healing of the fistula [8].

Surgery should be considered in all patients fit for surgery and remains the mainstay treatment as 75% of patients have septic complications [6]. The aim of surgery is to resect and anastomose the bowel segment as well as close the bladder defect. Treatment has radically changed over the years with single or staged procedures replacing palliative surgery with low mortality and improved quality of life. A single procedure involves resection and primary anastomosis of bowel and multi-stage procedures involve resection with primary anastomosis of bowel with stoma +/- Hartmann’s procedure (stage 2) with later stoma reversal (stage 3). The surgical approach must be individualized and is determined by the characteristics of the fistula, site of bowel lesion, underlying pathology, and patient’s pre-operative status. Primary anastomosis is performed in 95% of cases with no difference in complication rate [6]. Multi-stage procedures or preliminary defunctioning colostomy are limited to cases with high surgical risk, incurable malignant disease, presence of gross contamination or pelvic abscess, irradiated tissue, or extensive disease.

Laparoscopic surgery for diverticula-related colovesical fistula was first reported in 1994 [6,9] and is now the standard approach in most centers (75%) [6]. Although dense adhesions secondary to inflammation can make surgery technically demanding with an increased rate of conversion, it is minimally invasive with the advantages of early gut recovery, shorter hospital stay, and 27% lower post-operative morbidity rate [9]. Only 2% of presentations require emergency surgery [10]. Allowing the acute inflammation to resolve facilitates safe and successful single-stage surgery in 84% of cases [3,10]. A single-stage elective laparoscopic anterior resection and repair of colovesical fistula was performed electively two weeks later in this patient.

Opinion regarding treatment of the bladder defect is divided. Many techniques have been reported - Foley catheter drainage, simple closure of the fistula, omental patch closure, and partial or total cystectomy. Some small defects do not need closure. Studies have shown early removal of the urinary catheter does not increase morbidity, instead, long-term catheterization increases infection risk [6,10].

## Conclusions

Colovesical fistula may rarely present as epididymo-orchitis. The lack of symptoms and diagnostic challenges necessitate a high degree of suspicion to diagnose colovesical fistulas, especially in elderly patients with recurrent episodes of urinary tract infections without other underlying pathology.
